# Factors Affecting the Quality of Life and the Illness Acceptance of Pregnant Women with Diabetes

**DOI:** 10.3390/ijerph13010068

**Published:** 2015-12-22

**Authors:** Agnieszka Bień, Ewa Rzońca, Angelika Kańczugowska, Grażyna Iwanowicz-Palus

**Affiliations:** 1 Independent Obstetric Skills Unit, Faculty of Health Sciences, Medical University of Lublin, 4 Staszica St., 20-081 Lublin, Poland; eva.rzonca@gmail.com (E.R.); spupalus@gmail.com (G.I.-P.); 2 Neonatology Ward, Independent Public Teaching Hospital No. 4 in Lublin, 8 Jaczewskiego St., 20-954 Lublin, Poland; angelika_kanczugowska@wp.pl

**Keywords:** pregnancy, diabetes, quality of life, illness acceptance

## Abstract

The paper contains an analysis of the factors affecting the quality of life (QoL) and the illness acceptance of diabetic pregnant women. The study was performed between January and April, 2013. It included 114 pregnant women with diabetes, hospitalized in the High Risk Pregnancy Wards of several hospitals in Lublin, Poland. The study used a diagnostic survey with questionnaires. The research instruments used were: The WHOQOL-Bref questionnaire and the Acceptance of Illness Scale (AIS). The women’s general quality of life was slightly higher than their perceived general health. A higher quality of life was reported by women with a very good financial standing, very good perceived health, moderate self-reported knowledge of diabetes, and also by those only treated with diet and stating that the illness did not interfere with their lives (*p* < 0.05). Women with a very good financial standing (*p* < 0.009), high self-reported health (*p* < 0.002), and those treated with by means of a diet (*p* < 0.04) had a higher acceptance of illness. A higher acceptance of illness contributes to a higher general quality of life and a better perception of one’s health.

## 1. Introduction

Diabetes is one of the most frequent metabolic complications of pregnancy. It affects approx. 0.3% of women of reproductive age; in pregnant women the frequency is approx. 2%–6% [1]. Like other chronic illnesses, diabetes can adversely affect virtually all aspects of a patient’s life. It often leads to a deterioration in the patient’s physical and psychological wellbeing, a change in their lifestyle and its adaptation to the illness, as well as changes in physical, professional, and social activity and also values. All this also affects the patient's quality of life [2,3,4,5,6]. Gestational diabetes mellitus (GDM) is defined as any impairment of carbohydrate tolerance with onset or first recognition during pregnancy. Typical cases of GDM that subside after delivery are diagnosed between the 24th and the 28th week of pregnancy. Hyperglycemia observed in earlier gestational weeks may indicate previously undiagnosed type 2 diabetes mellitus or type 1 diabetes developing during the pregnancy. An initial fasting glucose level test to diagnose for gestational hyperglycemia should be ordered early in the pregnancy, during the first physician or midwife consultation. For pregnant women with risk factors, *i.e.*, pregnancy after the age of 35, history of large birth weight in previous pregnancies (>4000 g), delivery of infants with birth defects, history of fetal death, hypertension, overweight or obesity, family history of type 2 diabetes, GDM in previous pregnancies, or multiparity, the 75 g oral glucose tolerance test (OGTT) is required. If glycemia is normal, the test should be readministered at 24–28 weeks of pregnancy or when first symptoms indicative of diabetes are observed. For women without the risk factors, the 75g OGTT is administered at 24–28 weeks of pregnancy. Hyperglycemia during the pregnancy increases the risk of complications for the woman and for the developing fetus, and affects the child’s further development [1,3].

A diagnosis of diabetes during pregnancy is typically unexpected and may enhance negative experiences, changing the way the period of pregnancy is perceived. Furthermore, in pregnant women with diabetes, quality of life may be affected by worries about their health and that of the child, as well as by a feeling of losing control of one’s health [7,8,9,10,11].

Recently, an increased focus on health care costs and the assessment of treatment effectiveness has been observed, which contributes to increased interest in quality of life in medicine [4,12,13]. Currently, quality of life is a significant indicator of treatment effectiveness, used concurrently with clinical and functional assessment [5,14]. Quality of life is an interdisciplinary concept, with no single definition in the literature. Various views on quality of life have emerged in all fields of study related to human existence, such as medicine, philosophy, psychology, pedagogics, sociology, economics, and political science. As defined by the World Health Organization, quality of life comprises an individual’s perceptions of their own life in the context of their culture and value systems, and their personal goals, standards, and concerns [14,15,16]. 

Healthcare professionals regard quality of life as a reflection of an individual’s health, comprising their physical, psychological, and social wellbeing [14,17,18]. In 1997, Saxena and Orley [19] identified factors comprising an individual’s quality of life, based on the WHO definition. These were: Physical health, psychological condition, independence, relationships with others, and the environment one lives in. One other factor used in quality of life assessment is illness acceptance. Evaluating illness acceptance fits in with medical researchers’ generally increased interest in quality of life. This is due to shifts in medical ideology, which has acknowledged the need for a comprehensive assessment of patients’ health, including their living standards and social standing in their own life environment. Illness acceptance, or lack thereof, affects both quality of life and satisfaction with life in diabetic patients [6,20,21].

The main objective of the study was to analyze the factors affecting quality of life and illness acceptance in women diagnosed with diabetes during pregnancy.

## 2. Materials and Methods

### 2.1. Subjects

The study was performed between January and April 2013 among pregnant women with diabetes hospitalized in High Risk Pregnancy Wards at the following hospitals in Lublin, Poland: Independent Public Teaching Hospital no. 4, Independent Public Teaching Hospital no. 1, and the John of God Independent Public Regional Hospital. The inclusion criterion was diabetes first diagnosed during the pregnancy, in accordance with the current guidelines of the Polish Diabetology Society, *i.e.*, with fasting glucose levels of 92–125 mg/dL and/or a level of ≥180 mg/dL after 60 min and/or a level of 153–199 mg/dL after 120 min [22]. Women diagnosed with other conditions complicating the pregnancy, and which could have affected the subjects’ subjective quality of life and illness acceptance, such as: hypertension, imminent preterm birth, thyroid disease, liver disease *etc.*, were excluded from the study. The study was performed in accordance with the Helsinki Declaration, and was approved by the Ethical Committee of the Polish Midwives’ Association (II/EC/2012PMA). Before the questionnaires were completed, the hospital authorities’ permission was obtained. Respondents were informed that participation was voluntary, and that the study results were both anonymous and to be used exclusively for research purposes. 114 correctly completed questionnaires were received. The survey response rate was 87.7%.

### 2.2. Assessments

The study used a diagnostic survey with questionnaires. The research instruments used were: An own questionnaire based on the literature in the field, collecting participants’ socio-demographic data and assessing the influence of diabetes on various aspects of the participants’ lives; the WHOQOL-Bref (*World Health Organisation Quality of Life-Bref*) questionnaire; and the AIS (*Acceptance of Illness Scale*). 

The WHOQOL-Bref (*World Health Organisation Quality of Life-Bref*) questionnaire, assessing quality of life, is an abbreviated version of the WHOQOL-100 questionnaire. It consists of 26 questions, allowing an assessment of quality of life in four domains: physical, psychological, social, and environmental (each score reflects individual perception of quality of life in the given domain), as well as an assessment of the global quality of life and perceived general health of the patient. The questionnaire has undergone linguistic, cultural, and psychometric adaptation for use with Polish patients by Jaracz and Wołowicka [23]. WHOQOL-Bref is a research instrument enabling an assessment of quality of life both in healthy and ill individuals. The scores in each domain are determined by calculating means from all the items contained in a given domain. The scores in each domain may range from 4 to 20 points. The scores for general quality of life and perceived health, as well as individual domain scores, are positive, *i.e.*, higher scores indicate a higher quality of life. The questionnaire’s reliability, measured by the internal consistency coefficient (Cronbach’s α) is 0.54–0.91 for individual domains; for the whole scale it is 0.92 for healthy individuals, and 0.95 for ill individuals [23,24].

The AIS (*Acceptance of Illness Scale*) was developed by Felton, Revenson, and Hinrichsen, researchers from the Center for Community Research and Action, Department of Psychology, New York University. It has been adapted for use with Polish patients by Juczyński [25]. The scale may be used for any illness. It measures the level of illness acceptance in adult patients. Acceptance of one’s condition manifests itself in less intense reactions and sensations related to the illness and its treatment. The scale consists of eight statements describing the negative consequences of poor health. These are related to limitations resulting from one’s condition, lack of self-sufficiency, a feeling of being dependent on others, and lowered self-esteem. The more a patient accepts their illness, the better they adapt to the limitations resulting from the condition, and the less psychological discomfort they experience. A low score indicates no acceptance or adaptation to illness, as well as likely psychological discomfort, which may result in negative emotions. The respondents’ answers are scored 1–5 as follows: 1—strongly agree, 2—agree, 3—don’t know, 4—disagree, 5—strongly disagree. The total, between 8 and 40 points, is a measure of illness acceptance. Scores below 20 points are considered low, and indicate no or poor acceptance and adaptation to illness, and significant emotional problems related to it. Scores between 20 and 30 points indicate a moderate level of acceptance. Scores above 30 points indicate high or full acceptance of one's condition. The internal consistency coefficient—Cronbach’s α—is 0.85. The reliability of the Polish version of the AIS is close to that of the original, where Cronbach’s α was 0.82 [25].

### 2.3. Statistical Analyses

Statistical analysis was performed for the results obtained. The software used for databases and statistical analysis was STATISTICA 10.0 (StatSoft, Cracow, Poland). Quantitative parameters were presented using means, median values, and standard deviations; qualitative ones were presented as numbers and percentages. For quantitative parameters, normality was tested using the Shapiro-Wilk test. For the purpose of comparing two independent groups, the Mann-Whitney test was used. For comparing more than two groups, the Kruskal-Wallis test was used. Correlations between variables were measured using Spearman’s R. To find differences between the groups in terms of qualitative variables, the Chi-squared test was used. Correlations and differences at *p* < 0.05 were considered statistically significant.

## 3. Results

Among the 114 pregnant women studied, most were less than 30 years old (42.11%), college/university educated (64.91%), living in rural areas (35.09%), married (86.84%), reported their financial standing as good (52.63%), primigravid (38.60%), and in the third trimester of the pregnancy (76.31%)—Table 1. 

**Table 1:** Participants’ characteristics.

Socio-Demographic Data		*N*	%
Age	under 30 y/o	48	42.11
	30–35	39	34.21
	over 35 y/o	27	23.68
Education	primary	1	0.88
	vocational	8	7.02
	high school	31	27.19
	college/university	74	64.91
Residence	urban, above 100,000 residents	34	29.83
	urban, 50,000–100,000 residents	20	17.54
	urban, 50,000 residents	20	17.54
	rural	40	35.09
Marital status	single	10	8.77
	married	99	86.84
	divorced	4	3.51
	widow	1	0.88
Financial standing	very good	14	12.28
	good	60	52.63
	average/poor	40	35.09
Number of pregnancies	first pregnancy	44	38.60
	second pregnancy	40	35.09
	third pregnancy	22	19.30
	fourth or later pregnancy	8	7.01
Time in the pregnancy	first trimester	4	3.51
	second trimester	23	20.18
	third trimester	87	76.31

Table 2 shows how diabetes affected various aspects of the respondents’ lives. Most respondents reported good health (63.16%), moderate knowledge of diabetes (64.04%), some difficulties in everyday life due to the illness (42.98%), and being treated with a diet (63.16%).

**Table 2:** Influence of diabetes on various aspects of the respondents’ lives.

Aspect of Life		*N*	%
Self-reported health	very good	12	10.53
	good	72	63.16
	moderate/poor	30	26.31
Self-reported knowledge on diabetes	high	16	14.04
	moderate	73	64.04
	poor	25	21.92
Diabetes interferes with everyday life	yes	47	41.23
	no	18	15.79
	sometimes	49	42.98
Diabetes treatment method	diet	72	63.16
	diet and insulin	42	36.84

The women’s general quality of life was slightly higher than their perceived general health: 3.58 *vs.* 3.17. The respondents perceived their quality of life to be the best in the social, physical, and environmental domains, and slightly worse in the psychological domain (Table 3).

**Table 3:** Quality of life scores among the women studied.

Domains	Mean	Median (Me)	Min.	Max.	Standard Deviation (SD)
General quality of life	3.58	4.00	2.000	5.00	0.77
General health	3.17	3.00	1.000	5.00	0.84
Physical health	14.73	14.86	7.429	20.00	2.56
Psychological	13.87	14.00	8.000	18.67	2.21
Social relationships	15.40	16.00	6.667	20.00	2.49
Environment	14.10	14.25	7.500	19.50	2.08

Statistical analysis showed that respondents in a good or very good financial standing experienced a higher quality of life than those in an average or poor financial standing. The statistical analysis performed showed significant differences in quality of life scores in physical (*p* = 0.006), psychological (*p* < 0.0001) and environment domains (*p* < 0.0001). Those respondents who reported very good health enjoyed a significantly higher quality of life in all domains, and had a higher score in the perceived general health category, compared to other respondents (*p* < 0.05). Women who reported moderate knowledge of diabetes had a higher score in the perceived general health category, compared to other respondents (*p* < 0.02). Statistical analysis showed that respondents who stated that the illness does not interfere with everyday life experienced a significantly higher quality of life compared with those reporting that diabetes does interfere with everyday life some or most of the time. Differences were observed in terms of general quality of life (*p* = 0.006), perceived general health (*p* = 0.006), and quality of life in the physical (*p* = 0.003), psychological (*p* = 0.01), and environmental (*p* = 0.0001) domains. Pregnant women only treated with a diet enjoyed a higher quality of life than those treated with a diet and insulin. Statistical analysis showed significant differences between the two groups in terms of perceived general health (*p* = 0.01) and quality of life in the physical domain (*p* = 0.02) (Table 4).

The mean acceptance of illness score in the AIS scale within the studied group of diabetic women was 30.66 ± 7.08 (Me = 32.00). The greatest obstacles to the pregnant women’s acceptance of diabetes included: difficulties adjusting to the limitations of illness, limitations in doing one’s favorite activities, others’ discomfort around the patient due to the illness, and increased dependence on others (Table 5).

The study showed that women with a very good financial standing (*p* < 0.009), a very good perceived health score (*p* < 0.002), and treated with a diet (*p* < 0.04) had a higher acceptance of illness. Other socio-demographic characteristics (age, education, residence, marital status, number of pregnancies, and time in the pregnancy) or other aspects of life analyzed (self-reported knowledge on diabetes) had no influence on illness acceptance (*p* > 0.05) (Table 6).

**Table 4:** Quality of life scores in relation to socio-demographic data and various aspects of life.

Domains	Financial Standing																		Statistical Analysis	
	Very Good						Good						Average/Poor							
	Mean		Me		SD		Mean		Me		SD		Mean		Me		SD		H	*p*
General quality of life	3.79		4.00		0.80		3.67		4.00		0.73		3.38		4.00		0.81		3.72	0.16
General health	3.14		3.00		0.86		3.28		3.00		0.87		3.00		3.00		0.78		2.94	0.23
Physical health	15.76		16.29		2.27		15.18		15.14		2.39		13.70		13.71		2.60		10.21	0.006
Psychological	14.95		15.67		2.78		14.44		14.67		1.94		12.62		12.67		1.83		24.47	<0.0001
Social relationships	16.19		16.00		2.27		15.51		16.00		2.43		14.97		14.67		2.62		3.60	0.16
Environment	15.25		16.00		3.17		14.46		14.50		1.65		13.16		13.00		1.86		18.05	0.0001
**Domains**	**Self-Reported Health**																			
	**Very Good**						**Good**						**Moderate/Poor**						**H**	***p***
General quality of life	4.25		4.00		0.87		3.65		4.00		0.63		3.13		3.00		0.82		18.79	0.0001
General health	3.92		4.00		0.79		3.35		3.50		0.73		2.43		2.00		0.57		35.74	<0.0001
Physical health	17.24		16.57		1.44		15.12		14.86		2.12		12.80		12.86		2.61		30.32	<0.0001
Psychological	15.67		16.33		2.55		14.29		14.67		1.74		12.13		12.33		2.07		29.04	<0.0001
Social relationships	17.33		16.67		1.80		15.43		16.00		2.48		14.58		13.33		2.37		13.17	0.001
Environment	16.04		15.75		1.76		14.45		14.50		1.70		12.48		12.50		2.00		28.78	<0.0001
**Domains**	**Self-Reported Knowledge on Diabetes**																			
	**Poor**						**Moderate**						**High**						**H**	***p***
General quality of life	3.28		3.00		0.89		3.71		4.00		0.70		3.44		3.50		0,81		5.59	0.06
General health	2.84		3.00		0.80		3.33		3.00		0.80		2.94		3.00		0.93		7.21	0.02
Physical health	14.15		14.86		2.63		14.79		14.86		2.54		15.36		15.71		2.47		1.81	0.40
Psychological	13.04		14.00		2.07		14.12		14.00		2.08		14.00		14.67		2.79		4.45	0.11
Social relationships	15.41		16.00		2.91		15.27		14.67		2.41		16.00		16.00		2.18		1.48	0.48
Environment	13.54		14.00		2.47		14.18		14.00		1.81		14.63		15.00		2.47		3.46	0.18
**Domains**	**Diabetes Interferes with Everyday Life**																		**H**	***p***
	**Yes**						**No**						**Sometimes**							
General quality of life	3.32		3.00		0.75		3.94		4.00		0.73		3.69		4.00		0.74		10.27	0.006
General health	2.91		3.00		0.78		3.61		4.00		0.85		3.24		3.00		0.83		10.12	0.006
Physical health	13.65		13.71		2.36		16.06		16.86		2.97		15.28		16.00		2.19		15.92	0.003
Psychological	13.16		13.33		2.05		14.48		14.67		2.20		14.31		14.67		2.22		8.52	0.01
Social relationships	14.81		14.67		2.71		15.56		15.33		2.29		15.92		16.00		2.25		3.74	0.15
Environment	13.14		13.50		1.94		15.28		15.00		1.93		14.59		14.50		1.89		18.77	0.0001
**Domains**	**Diabetes Treatment Method**																		**Z**	***p***
	**Diet**									**Diet and Insulin**										
	**Mean**			**Me**			**SD**			**Mean**			**Me**			**SD**				
General quality of life	3.67			4.00			0.79			3.43			4.00			0.74			1.52	0.13
General health	3,33			3.00			0.79			2.88			3.00			0.86			2.49	0.01
Physical health	15.16			15.43			2.52			14.00			13.71			2.48			2.40	0.02
Psychological	13.97			14.00			2.22			13.68			14.00			2.21			0.66	0.51
Social relationships	15.50			16.00			2.60			15.24			16.00			2.30			0.43	0.67
Environment	14.26			14.00			1.84			13.82			14.50			2.43			0.31	0.76

**Table 5:** Average acceptance of illness scores in the AIS scale within the group of diabetic women studied.

Statement	Score				
	Mean	Me	Min.	Max.	SD
I have a hard time adjusting to the limitations of my illness	3.07	3.00	1.00	5.00	1.16
Because of my health, I miss the things I like to do most	3.46	4.00	1.00	5.00	1.24
My illness makes me feel useless at times	4.25	5.00	1.00	5.00	1.22
Health problems make me more dependent on others than I want to be	3.71	4.00	1.00	5.00	1.34
My illness makes me a burden on my family and friends	4.16	5.00	1.00	5.00	1.29
My health makes me feel inadequate	4.11	5.00	1.00	5.00	1.29
I will never be self sufficient enough to make me happy	4.24	5.00	1.00	5.00	1.19
I think people are often uncomfortable being around me because of my illness	3.67	4.00	1.00	5.00	1.38
Total AIS score	30.66	32.00	11.00	40.00	7.08

**Table 6:** AIS scores in relation to socio-demographic data and various aspects of life.

Socio-Demographic Data	Mean	SD	Me
**Financial standing**			
very good	33.36	6.97	35.00
good	31.58	6.78	33.00
average/poor	28.33	7.08	29.00
Statistical analysis: *H* = 9.46; *p* = 0.009			
**Self-reported health**			
very good	35.17	6.10	37.50
good	30.90	6.98	32.00
moderate/poor	28.27	6.89	28.50
Statistical analysis: *H* = 12.16; *p* = 0.002			
**Diabetes treatment method**			
diet	31.85	6.30	33.00
diet and insulin	28.62	7.92	29.00
Statistical analysis: *Z* = 2.06; *p* = 0.04			

An analysis of correlations between illness acceptance and quality of life showed significant correlations between illness acceptance and scores in the general quality of life (*R* = 0.39) and perceived general health (*R* = 0.39) categories. Significant correlations were also found between illness acceptance and all specific quality of life domains (*p* < 0.05). The strength of correlations was rated between 0.20 and 0.54 (Table 7). 

**Table 7:** Correlation between the acceptance of illness (AIS) and quality of life (QoL) scores.

Domains	Statistical Analysis	
	*R*	*p*
General quality of life	0.39	0.00002
General health	0.39	0.00002
Physical health	0.54	<0.00001
Psychological	0.40	0.00001
Social relationships	0.20	0.03
Environment	0.42	<0.00001

## 4. Discussion

Quality of life studies allow the identification of optimal treatment and care methods based on a given patient's life situation and biopsychosocial status [2,14]. The present paper attempted to analyze the factors affecting quality of life and illness acceptance in women diagnosed with diabetes during pregnancy. The influence of socio-demographic factors and selected aspects of everyday life (including self-reported health, self-reported knowledge on the illness, diabetes’ interference with everyday life, financial standing, and treatment methods) was studied. Diabetes significantly affects the patient’s lifestyle, psychological comfort, and wellbeing, contributing to increased sensitivity, a sense of losing control, increased stress, and may undermine quality of life [5,9,10,14,26,27]. The results of the DAWN (*Diabetes Attitudes, Wishes, and Needs*) study showed that approximately 50% of diabetic patients have a lowered quality of life, and that diabetes contributes to numerous psychosocial problems [11]. Studies by Hjelm *et al.* [28], Mezuk *et al.* [29] and Kozhimannil *et al.* [30] indicate that the diagnosis of gestational diabetes may increase the risk of depression, due to worries about one’s own and the child’s health and also because of the awareness of lifestyle changes necessary to reduce the risk of developing type 2 diabetes. 

The authors’ studies showed that women’s general quality of life was slightly higher than their perceived general health. This is also confirmed by Mortazavi *et al.* [31]. Results by Dalfrà *et al.* [8] show that women with gestational diabetes mellitus (GDM) have higher scores in the perceived general health category than pregnant women with type 1 diabetes, but lower than healthy pregnant women. Studies by Runbold and Crowther [32] and Nolan *et al.* [7] indicate that for women with GDM, the diagnosis adversely affects their perception of their own health. Results by Kim and Vahratian [33] indicate that the GDM diagnosis may increase anxiety, contribute to a negative perception of one’s health, and interfere with the positive experiences of pregnancy in comparison to women with a normal course of pregnancy.

A low quality of life may interfere with achieving therapeutic objectives. Patients who do not know the strategy of diabetes management may feel overwhelmed and exhausted by the disease, which lowers their quality of life. Thus, they become less involved in the therapeutic process, which contributes to negligence in self-control, and as a consequence, this increases the risk of complications and further deterioration of quality of life. Therefore, increasing quality of life is one of the primary objectives in diabetes treatment, aside from achieving metabolic balance and preventing complications [5]. 

The quality of life assessment performed by the authors focused on the individual’s functioning in the physical, psychological, and social domains, as well as their functioning in their environment. With regard to these domains, the respondents experienced the highest quality of life in the social and physical domains. The present results are corroborated by those obtained by Zubaran *et al.* [34] and, in part, by those obtained by Mortazawi *et al.* [31], where the pregnant women reported the highest quality of life in the environment and social domains. 

One obstacle to independence in one’s struggle with a chronic illness is the patient’s financial standing [4,5,35]. The authors’ analysis showed that a very good financial standing reported contributed to a higher quality of life in the physical, psychological, and environmental domains. Financial means are an important factor in the patient’s health—diabetes is related with increased spending on medicines, tests, and food, affecting the patient’s family finances and their psychological state, as emphasized in studies by Hjelm *et al.* [35] and Fort *et al.* [4]. This is not, however, confirmed by Lapolla *et al.* [11], reporting that treatment costs are not a burden on family finances.

Danyliv *et al.* [36] report that in a group with normal glucose tolerance, socio-economic factors influence self-reported health-related quality of life (HRQoL). A higher income meant a higher HRQoL—a relationship not demonstrated in the GDM group. Surprising results indicating the contrary were reported by Felicio *et al.* [37]. In their study, the highest HRQoL was reported by participants of low or very low economic status, with the lowest monthly incomes [37]. The authors’ own results show that the women’s average or poor financial standing correlated with worse quality of life scores in the physical, psychological, and environmental domains.

The authors’ analysis showed that better self-reported health was correlated with higher quality of life scores in the four domains assessed, as well as with higher scores in the general quality of life and perceived general health categories. In another study, Hjelm *et al.* [35] showed that women with GDM reported beliefs and attitudes related to health, illness, and care based on the health care unit used, health care professionals’ beliefs, and educational model. At this point, one should emphasize the necessity of raising the patients’ awareness in relation to health through well-organized health care provided by qualified and knowledgeable professionals [10,35,38].

The authors’ own results show that scores in the perceived general health category were higher for those women who reported their level of knowledge of diabetes as moderate. Patients with poor health awareness are more likely to report their health as poor, to have a low level of education (therefore, to lack knowledge of their disease and treatment), and a low socio-economic status [39,40,41,42]. However, results by Evans and O’Brien [43] should be considered as well. These indicate that for some pregnant women knowledge of diabetes increases motivation and the sense of one’s own capability to make lifestyle changes. Hjelm *et al.* [28] reported that beliefs on health and illness correlate with risk awareness and self-care capabilities. The Swedish women in the study intended to combat their illness and undertook long-term efforts to do so; meanwhile, Middle Eastern women adjusted to their GDM, and considered it a natural part of their lives rather than a health problem related to pregnancy.

The authors’ studies show that a higher quality of life was also reported by those pregnant women who were only treated with a diet. Significant differences were found between patients only treated with a diet and those treated with a diet and insulin in terms of perceived general health and quality of life in the physical domain. Dalfrà *et al.* [8], however, state that pregnant women treated with insulin had lower stress levels than those only treated with a diet. One possible explanation is that the achievement of good metabolic control through insulin treatment may be calming, as patients are aware that metabolic balance is important both for them and for their child. Nonetheless, one must remember that women requiring insulin treatment may also need additional support in adjusting to the diagnosis [10].

The further part of the paper contains an analysis of illness acceptance among pregnant women with diabetes. Study results show that the mean illness acceptance score in the sample of pregnant women studied was 30.66 points, which indicates high or full acceptance of one's condition. These results are higher than those obtained by other researchers in groups of diabetic patients—in the study by Niedzielski *et al.* [44], the mean acceptance of illness was 23.33, and in the study by Lewko *et al.* [45], it was 29.6. It is likely that a longer duration of the illness and the resulting limitations contribute to the lower acceptance.

The analysis of the results obtained showed that the lowest mean score, and therefore, the lowest level of illness acceptance, was obtained for the statement *“I have a hard time adjusting to the limitations of my illness”* (mean 3.07; SD 1.16), whereas the highest mean score and the highest level of adjustment to illness was related to the denial of the statement “*My illness makes me feel useless at times”* (mean 4.25; SD 1.22). The necessity of hospitalization due to diabetes diagnosis, and learning about diabetes management, was a source of significant discomfort for the pregnant women studied, as was the necessity to adjust to the limitations imposed by the illness, and *“missing the things one likes to do most”*. On the other hand, the diagnosis of diabetes, despite the limitations it imposes on the respondents, does not make them feel useless or dependent.

In the next stage of the study, an attempt was made to determine whether socio-demographic factors and various aspects of life affect illness acceptance. The respondents with a very good financial standing (mean 33.36) obtained significantly higher scores in the AIS than those who reported an average or poor financial status (mean 28.33), which may indicate that sufficient financial means make one more comfortable in the treatment process. Financial difficulties may interfere with treatment, limit access to healthy nutrition, and even disrupt the everyday life of a family [4].

Acceptance is a very important stage in the patient's relation to the illness. It facilitates adaptation, that is, the process whereby the patient adjusts to their new situation—living with the illness. Acceptance of illness gives the patient a sense of security, increases trust towards physicians and treatment methods, favors the patient’s active participation in treatment, and instills an optimistic and hopeful attitude towards life [45]. Studies by Lewko *et al.* [20] indicate that quality of life related to general health is positively correlated to the acceptance of illness scale, *i.e.*, a higher score indicates both a higher quality of life and a higher acceptance of diabetes in the group studied. Similar results were obtained in the present study. A higher level of illness acceptance in the group corresponded to a higher quality of life in all domains and a better perceived general health. The acceptance of limitations resulting from diabetes contributes to better motivation, goal achievement, and thus to overcoming the difficulties of illness. Patients who know and accept their illness are more motivated to overcome difficulties, and are more active in challenging situations. 

Quality of life assessment gives one a broader perspective on the patient, which is crucial for balancing the rigorous efforts aiming to achieve metabolic balance and normalization with the patient’s happiness and quality of life [2]. Quality of life assessment should become a routine part of diabetes care. It allows the patients’ stress, limitations in everyday life, and burdens resulting from treatment to be checked. This information is necessary for a full assessment of the patient’s condition, and is a factor in treatment. It enables the comprehensive understanding of the patient’s situation, the development of new methods of treatment and education, and the evaluation of these methods’ effectiveness [5,12].

By considering the impact of diagnosis on the quality of life and the experiences of pregnant women with diabetes, one may verify the patient's stress, limitations, and burdens experienced, and identify priorities in care [5,10]. This, in turn, may enhance the care and education provided to these women, and contribute to the promotion of self-control during pregnancy. It should also be emphasized that knowledge on the subjective quality of life of a given patient with GDM may optimize the scope and quality of care provided by health care professionals, physicians, and midwives, thus better satisfying the expectations of the patient.

## 5. Conclusions

Factors that affect the reported quality of life of pregnant women with diabetes are: the patients’ financial status, self-reported health, knowledge on the illness, difficulties in everyday life due to diabetes, and the method of treatment. 

Acceptance of illness among women diagnosed with diabetes during the pregnancy is affected by their financial status, self-reported health, and treatment method.

A higher acceptance of illness contributes to a higher general quality of life and a better perception of one’s health.
